# Validation of an Accurate Automated Multiplex Immunofluorescence Method for Immuno-Profiling Melanoma

**DOI:** 10.3389/fmolb.2022.810858

**Published:** 2022-05-19

**Authors:** Zarwa Yaseen, Tuba N. Gide, Jordan W. Conway, Alison J. Potter, Camelia Quek, Angela M. Hong, Georgina V. Long, Richard A. Scolyer, James S. Wilmott

**Affiliations:** ^1^ Melanoma Institute Australia, The University of Sydney, Sydney, NSW, Australia; ^2^ Charles Perkins Centre, The University of Sydney, Sydney, NSW, Australia; ^3^ Faculty of Medicine and Health, Sydney Medical School, The University of Sydney, Sydney, NSW, Australia; ^4^ Royal Prince Alfred Hospital and NSW Health Pathology, Sydney, NSW, Australia; ^5^ Faculty of Medicine and Health, University of New South Wales, Sydney, NSW, Australia; ^6^ GenesisCare, Radiation Oncology, Mater Hospital, Sydney, NSW, Australia; ^7^ Royal North Shore Hospital, Sydney, NSW, Australia; ^8^ Mater Hospital, Sydney, NSW, Australia

**Keywords:** multiplex immunofluorescence, immunohistochemistry, multispectral imaging, melanoma, tumour microenvironment, pathology, immunotherapy, predictive biomarker

## Abstract

Multiplex immunofluorescence staining enables the simultaneous detection of multiple immune markers in a single tissue section, and is a useful tool for the identification of specific cell populations within the tumour microenvironment. However, this technology has rarely been validated against standard clinical immunohistology, which is a barrier for its integration into clinical practice. This study sought to validate and investigate the accuracy, precision and reproducibility of a multiplex immunofluorescence compared with immunohistochemistry (IHC), including tissue staining, imaging and analysis, in characterising the expression of immune and melanoma markers in both the tumour and its microenvironment. Traditional chromogenic IHC, single-plex immunofluorescence and multiplex immunofluorescence were each performed on serial tissue sections of a formalin-fixed paraffin-embedded (FFPE) tissue microarray containing metastatic melanoma specimens from 67 patients. The panel included the immune cell markers CD8, CD68, CD16, the immune checkpoint PD-L1, and melanoma tumour marker SOX10. Slides were stained with the Opal^™^ 7 colour Kit (Akoya Biosciences) on the intelliPATH autostainer (Biocare Medical) and imaged using the Vectra 3.0.5 microscope. Marker expression was quantified using Halo v.3.2.181 (Indica Labs). Comparison of the IHC and single-plex immunofluorescence revealed highly significant positive correlations between the cell densities of CD8, CD68, CD16, PD-L1 and SOX10 marker positive cells (Spearman’s rho = 0.927 to 0.750, *p* < 0.0001). Highly significant correlations were also observed for all markers between single-plex immunofluorescence and multiplex immunofluorescence staining (Spearman’s rho >0.9, *p* < 0.0001). Finally, correlation analysis of the three multiplex replicates revealed a high degree of reproducibility between slides (Spearman’s rho >0.940, *p* < 0.0001). Together, these data highlight the reliability and validity of multiplex immunofluorescence in accurately profiling the tumour and its associated microenvironment using FFPE metastatic melanoma specimens. This validated multiplex panel can be utilised for research evaluating melanoma and its microenvironment, such as studies performed to predict patient response or resistance to immunotherapies.

## Introduction

Immunohistochemistry (IHC) is a clinical tool routinely used to diagnose cancer and its use is rapidly growing to phenotype and quantify immune infiltrates in cancer biopsies. Visualisation of the antibodies bound to antigens of interest is traditionally achieved through either chromogenic or fluorescent reporters bound to the secondary antibodies that detect the species-specific primary antibody (Kalra and Baker, 2017). However, the need for co-localisation of multiple markers, particularly for accurate immune phenotyping, has led to the development of multiple analyte platforms whereby multiple fluorophores or chromogens can be analysed on a single tissue section have allowed investigators to examine the expression of multiple targets of interest simultaneously on a single cell. This is especially useful in characterising the tumour immune microenvironment in cancer patients by the simultaneous detection of the location and interaction of immune cell subpopulations determined by their expression of immune markers (Hanahan and Weinberg, 2011; Teng et al., 2015). Likewise, the expression of biomarkers, such as the programmed cell death ligand 1 (PD-L1), are increasingly used to predict the likelihood of patient responsiveness to treatment, with co-localisation of markers aiding in the phenotyping of the cells expressing the biomarker (Herbst et al., 2014). Given the recent renewed research focus on the tumour microenvironment (TME), along with the role of immune markers as predictors of response to targeted immunotherapy for cancer patients, multiplex IHC is a useful and powerful tool in the analysis of the tumour microenvironment for both clinical and research purposes (Mori et al., 2020). In contrast to other tissue-based techniques used to quantify immune and tumour cells, such as flow cytometry and RNA sequencing (RNAseq), multiplex immunofluorescence has the advantage of preserving the integrity and histological location of immune, resident and tumour cells in tissue sections, as opposed to cell suspensions, tissue digestion, and homogenisation *via* flow cytometry and RNAseq techniques (Lee et al., 2020). This crucial distinction preserves the tissue architecture, facilitates the precise localisation of immune cell subsets and their spatial relationships with other cells, and allows an integral examination of the TME (Halse et al., 2018). The ability to simultaneously detect and localise multiple cells *in situ* depends on the sensitivity and reproducibility of the multiplex IHC staining workflow.

The TSA Opal multiplex immunofluorescence protocol is based on tyramide signal amplification (TSA) whereby a fluorescent Opal dye is conjugated with tyramide molecules and produces an enzymatically amplified signal. The protocol allows for the detection of up to six different markers along with a nuclear counterstain on a single tissue section involving sequential rounds of antibody stripping (Stack et al., 2014). This platform overcomes the limitations of traditional chromogenic IHC by allowing the detection of biomarkers that exhibit low expression along with the use of antibodies raised in the same species (Tóth and Mezey, 2007). IHC workflows have also notably improved with the development of assay automation, standardised whole-slide scanning, and image processing, reducing the likelihood of batch effects and human errors (Blom et al., 2017; Lim et al., 2018; Parra et al., 2020). Validating automated multiplex immunofluorescence renders the panels potentially useful for clinical practice (Lim et al., 2018).

Multiplex immunofluorescence staining is an important tool used to characterise the TME and use information about the cellular components to guide researchers and clinicians in understanding the biology of melanoma and improve treatment. The multiplex panel was designed as a 6-plex assay consisting of the following markers: immune cell markers CD8, CD68, CD16a, immune checkpoint PD-L1, melanoma tumour marker SOX10, and DAPI. CD8^+^ T cells play a paramount role in the anti-tumour response, with their presence in the TME correlated to improved survival in many cancers (Attrill et al., 2021). Location of these CD8 cells are important as current studies suggest that the presence of CD8^+^ cells in tumour-infiltrating lymphocytes (TILs) are associated with greater access to tumour and improved survival (Ling et al., 2007). Macrophages are seen to be involved in all facets of tumour progression along with playing a role in resistance to therapies (Aras and Zaidi, 2017). While the link between macrophage marker CD68 and outcome has been ambiguous, CD68^+^ cell counts located at the invasive front of the tumour has been noted to be a predictor of reduced survival (Piras et al., 2005). Furthermore, CD68^+^ macrophage infiltration affects gene expression within the tumour which impact cell processes like cell death and cell cycle (Tremble et al., 2020). The activation marker CD16 expressed by natural killer cells were found to be more responsive to cytokines produced by melanoma (Ali et al., 2014). The immune checkpoint PD-L1 has been found to be upregulated by tumours and concentrated at the tumour margins neighbouring CD8^+^ T cells (Tumeh et al., 2014). PD-L1 is the ligand of PD-1 which is upregulated by exhausted T cells and reduces T-cell effector functions upon binding with its ligand (Parry et al., 2005). SOX10 is a transcription factor that plays an important role in characterising neural crest cell and is a reliable, sensitive, and specific nuclear marker used for the detection of metastatic melanoma (Pytlak et al., 2019).

In this study, we sought to validate multiplex immunofluorescence digital pathology analysis by comparing the results obtained for immune and melanoma marker phenotype quantification by multiplex immunofluorescence, single-plex immunofluorescence, and traditional chromogenic immunohistochemistry. Using this data, we determined the robustness of an optimised multiplex IHC protocol in producing comparable, reproducible, and potentially clinically translational data using the multiplex platform.

## Materials and Methods

### Patient Cohorts and Specimens

Treatment-naïve melanoma patients with regional lymph node metastases available formalin-fixed paraffin-embedded (FFPE) specimens were identified from the MIA Melanoma Biospecimen Bank and acquired from the department of Tissue Pathology and Diagnostic Oncology at the Royal Prince Alfred Hospital, Sydney, Australia, with informed consent and Human Ethics Review Committee approval (Sydney Local Health District Ethics Review Committee Protocol No. X15-0454 and HREC/11/RPAH/444). A tissue microarray (TMA) was constructed with 1 mm^2^ tissue cores from 67 patients using a TMArrayer (Pathology Devices). Regions of metastasis consisting of high tumour content with high tumour infiltrating lymphocytes (TILS) were selected upon preparation of the TMA block using H&E sections from the whole tissue block to ensure the presence of melanoma cells in each sample. Five cores were excluded from all analyses due to loss of tissue integrity during staining. Furthermore, additional cores may have been excluded from specific staining categories due to loss of tissue integrity but were included in the analysis in other staining categories where the tissue integrity was preserved. The number of samples included in each analysis are noted in the figures and the corresponding figure legends.

### Tissue Preparation for Staining

Prior to staining, 4 µm sections of the TMA and a lymph node containing metastatic melanoma were cut on the Leica RM2125 RTS microtome, mounted on SuperFrost^™^Plus slides (Thermo Fisher Scientific), and dried over-night at room temperature. Upon sectioning the TMA, the first three adjacent slides were designated for CD8 staining (IHC slide, Single-plex slide, Single-plex without nuclear stain slide for spectral library preparation). The following slides were similarly designated in a sequential manner for markers CD68, CD16, PD-L1, SOX10, and DAPI/Hematoxylin (Table 1). The last five slides were designated for three multiplex stainings, a positive control slide, and a negative control slide respectively. Upon sectioning the lymph node containing metastatic melanoma, the first two slides were designated for CD staining (IHC slide and Single-plex control slide). The following slides were similarly designated in a sequential manner for markers CD68, CD16, PD-L1, and SOX10. The last two slides were designated for a positive control multiplex slide and a negative control multiplex slide respectively. All slides were deparaffinised by baking for 30 min at 65°C in a Dehydrating Oven (Thermoline Scientific) and were placed in xylene. Rehydration was performed through decreasing graded alcohol. Antigen retrieval was performed in high pH HIER buffer (pH 9) in the Decloaking Chamber (Biocare Medical) at 110°C for 10 min. Slides were cooled on the benchtop in TBST for 5 min before commencing staining. All staining was performed on the intelliPATH autostainer (Biocare Medical) at room temperature. Traditional chromogenic single-plex IHC staining was conducted in a separate staining run to the Opal single-plex and multiplex IHC staining.

**TABLE 1:** Six-plex immunofluorescence panel to phenotype melanoma and its tumour microenvironment in FFPE specimens.

Biomarker	Clone	Host species	IHC concentration	Opal dilution	Fluorophore	Position in multiplex	Vendor
CD68	KP-1	Mouse	1:200	1: 500	Opal 520	1	Cell Marque
CD8	C8/144B	Mouse	1:100	1:1500	Opal 570	5	Dako
CD16a	EPR16784	Rabbit	1:500	1:400	Opal 620	2	Abcam
PD-L1	E1L3N	Rabbit	1:200	1:1000	Opal 650	4	Cell Signalling
SOX10	BD34	Mouse	1:100	1:200	Opal 690	3	Biocare
—	—	—	—	—	Spectral DAPI	—	—

### Traditional Chromogenic Single-Plex Immunohistochemistry

Tissue sections underwent an endogenous peroxidase blocking step with 3% H_2_O_2_ for 5 min. The slides were incubated with either CD8 (Dako, clone C8/144B, mouse, dilution 1:100), CD68 (Cell Marque, clone KP-1, mouse, dilution 1:200), CD16a (Abcam, clone EPR16784, rabbit, dilution 1:500), PD-L1 (Cell Signalling Technology, clone E1L3N, rabbit, dilution 1:200), or SOX10 (Biocare Medical, clone BD34, mouse, dilution 1:100) for 45 min. Antibodies were diluted in Da Vinci Green (Biocare Medical). Antibodies were detected using the MACH3 detection kit (Biocare Medical) before visualisation with the Betazoid DAB chromogen Kit (Biocare Medical). The slides were counterstained with haematoxylin and then coverslipped with xylene. Whole sections of the lymph node containing metastatic melanoma were stained with each primary antibody and were used as positive controls. TMA section without primary antibody treatment was used as a negative control. All markers stained with chromogenic immunohistochemistry were reviewed by a pathologist to confirm staining patterns were consistent with known distributions.

### Panel Optimisation

Antibodies were validated by following the manufacturer’s recommendations for each antibody and consistent chromogenic immunohistochemistry staining comparable to manufacturer expectations were obtained. Opal-antibody pairings were designated taking into consideration antibody co-expression and relative abundance by pairing co-expressing antibodies to fluorophores spectrally distant. The position of each antibody in the panel was decided by placing the antibody in several positions and assessing staining quality and intensity. Ideal staining was defined by the absence of bleed-through into adjacent channels. Optimal antibody concentration was based on at least a 10-fold signal-to-noise ratio (Supplementary Table S1).

### Single-Plex Immunofluorescence Staining

The TSA-based Opal protocol was used for single-plex and multiplex immunofluorescence staining (Opal 7-Color Manual IHC Kit, Akoya Biosciences, Product number NEL801001KT). Six adjacent TMA sections and six lymph node sections were deparaffinised, antigen retrieved and blocked for endogenous peroxidase, as described above. Each TMA section underwent antigen retrieval and respective antibody staining simultaneously with the multiplex TMA slide according to its position in the multiplex panel. Tissue sections were blocked with 3% hydrogen peroxide in TBST for 5 min, and then incubated with either CD8 (Dako, clone C8/144B, mouse, dilution 1:1500), CD68 (Cell Marque, clone KP-1, mouse, dilution 1:500), CD16a (Abcam, clone EPR16784, rabbit, dilution 1:400), PD-L1 (Cell Signalling Technology, clone E1L3N, rabbit, dilution 1:1000), or SOX10 (Biocare Medical, clone BD34, mouse, dilution 1:200) for 30 min. Antibodies were diluted in Da Vinci Green (Biocare Medical). The antibody was detected using the Opal Polymer HRP Ms + Rb (Onestep) (AKOYA Biosciences) detection system before visualisation using the respective Opal TSA (1:100) for another 5 min. Slides were counterstained with DAPI (1:2000) for nuclei visualisation for 5 min and coverslipped using the ProLong^®^ Diamond Antifade Mountant (Invitrogen). Whole sections of a lymph node containing metastatic melanoma were stained with each respective primary antibody alongside each TMA single-plex and were used as positive controls. Separate TMA sections without primary antibody treatment were used as negative controls alongside each TMA single-plex.

### Multiplex Immunofluorescence Staining

Four adjacent TMA sections and one whole section of a lymph node containing metastatic melanoma were deparaffinised, antigen retrieved and blocked for endogenous peroxidase as described before. Tissue sections were blocked with 3% hydrogen peroxide in TBST for 5 min, and then incubated with the antibody for CD68 (Cell Marque, clone KP-1, mouse, 1:500) for 30 min. The antibody was detected using the Opal Polymer HRP Ms + Rb (Onestep) (AKOYA Biosciences) detection system, before visualisation using Opal520 TSA (1:100) for another 5 min. Subsequently, antigen retrieval was conducted again to prepare the slides for the next antibody. Antibodies were diluted in Da Vinci Green (Biocare Medical). Using this method, all samples were stained sequentially with CD16a (Abcam, clone EPR16784, rabbit, dilution 1:400) visualised with Opal620 TSA (1:100), SOX10 (Biocare Medical, clone BD34, mouse, dilution 1:200) visualised with Opal690 TSA (1:100), PD-L1 (Cell Signalling Technology, clone E1L3N, rabbit, dilution 1:1000) visualised with Opal650 TSA (1:100), and CD8 (Dako, clone C8/144B, mouse, dilution 1:1500) visualised with Opal570 TSA (1:100). Slides were counterstained with DAPI (1:2000) for nuclei visualisation for 5 min and coverslipped using the ProLong^®^ Diamond Antifade Mountant (Invitrogen). Whole sections of a lymph node containing metastatic melanoma were stained simultaneously with each respective primary antibody alongside the TMA multiplex and was used as a positive control. A TMA section without primary antibody treatment was used as a negative control alongside the TMA multiplex. All markers stained with multiplex immunofluorescence were reviewed by a pathologist.

### Image Analysis

All immunohistochemistry and immunofluorescence slides were scanned using the Vectra 3.0 and images visualized in Phenochart v.1.0.8 (AKOYA Bioscience). Before scanning the slides, optimal scanning protocols were created by optimising the exposure time for each filter cube at ×10 and ×20 magnification. A ×10 objective lens was used for whole slide scans while a ×20 objective lens was used to capture high resolution images of each core tissue region. For the immunofluorescence slides, whole-slide images were scanned with all five, standard epi-fluorescence filters (DAPI, FITC, Cy3, Texas Red and Cy5). A spectral library for each fluorophore was generated in inForm v2.4.1 (AKOYA Biosciences) by using snapshots of representative tissue areas from the single-plex TMA slide stained for each single fluorophore without DAPI staining. All TMA core images were then spectrally unmixed using the spectral libraries. For the immunohistochemistry slides, whole-slide images were scanned in bright field. A library for haematoxylin and DAB was generated in inForm v2.4.1 (AKOYA Biosciences) using representative tissue areas from haematoxylin-only and DAB-only slides. TMA core images were analysed in HALO v2.2 (Indica Labs). The Random Forest tissue classifier was used to train the algorithm on multiple tissue areas to recognise the tissue and slide regions. Positivity for each individual marker was determined by the intensity of the staining with a minimum of a 10-fold signal-to-noise ratio (Supplementary Table S1), the staining pattern in accordance with the manufacturer’s expectations and previous literature, and comparable staining with the control tissue. Analysis settings were created for each staining category by optimising the thresholds and running the analysis on all samples. Upon revision of analysis performance, tailored analysis settings were created for select samples to account for tissue and staining variability. Staining artifacts, including tissue folds, tears and pigment accumulation, were excluded from analysed regions (Supplementary Figure S1).

**FIGURE 1:**
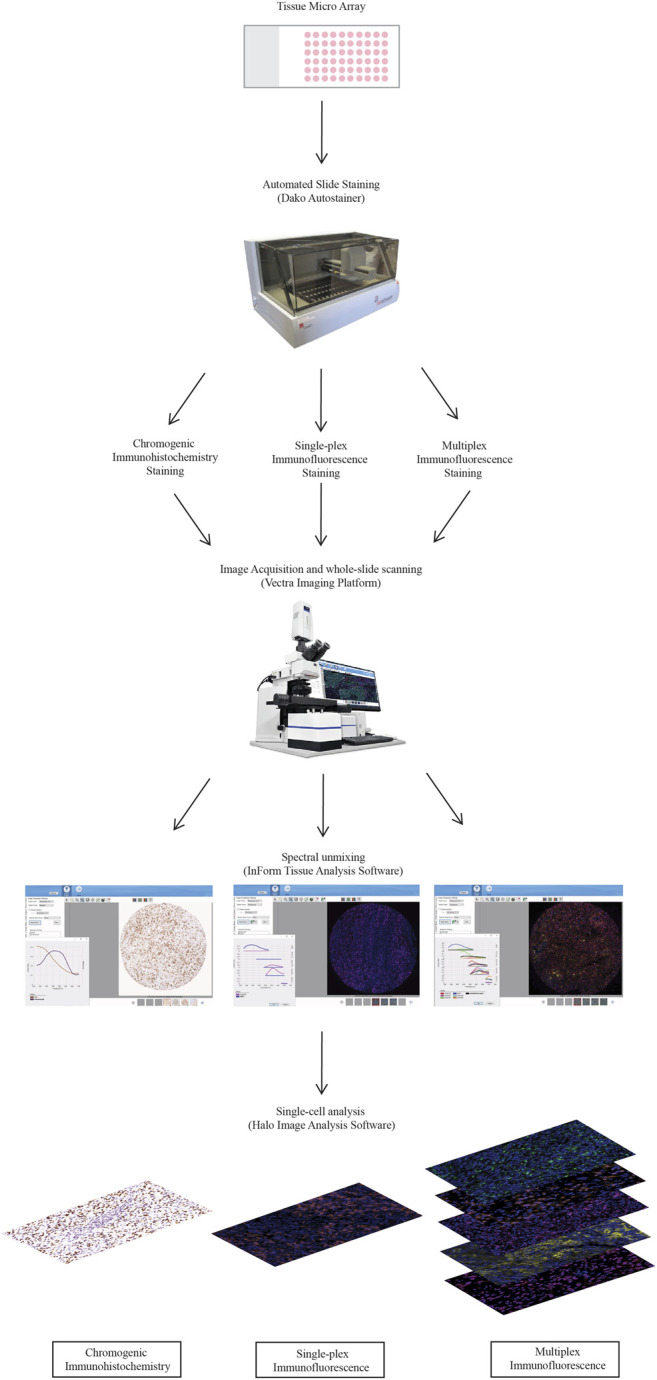
Workflow for traditional chromogenic and immunofluorescence immunohistochemistry representing the steps taken from staining the TMA slides to image acquisition and analysis. TMA slides were stained on the Dako autostainer and then stained with chromogenic immunohistochemistry, single-plex immunofluorescence and multiplex immunofluorescence. Stained whole-slides were scanned with the Vectra 3.0 and high resolution images (20×) were acquired. Images were spectrally unmixed using inform. Analysis at the single-cell level was conducted using Halo Image Analysis Software.

A diagrammatical representation of the workflow from the staining to the analysis is summarised in Figure 1.

### Statistical Analysis

All statistical analyses were conducted using PRISM version 8 (Graphpad Software, San Diego, CA, United States). The Spearman’s rank correlation was performed between traditional chromogenic IHC and immunofluorescence (single-plex and multiplex) for each marker. A *p*-value of less than 0.05 was considered statistically significant unless otherwise stated. To compare the staining intensity of the samples stained with single-plex and multiplex immunofluorescence, one-way analysis of variance (ANOVA) with Tukey’s HSD (honestly significant differences) test was performed to evaluate the staining intensity differences between specific groups (i.e. Single-plex, Multiplex 1, 2 and 3) with each marker.

## Results

### Traditional Single Chromogenic IHC Staining Correlates With Single-Plex Immunofluorescence

**FIGURE 2:**
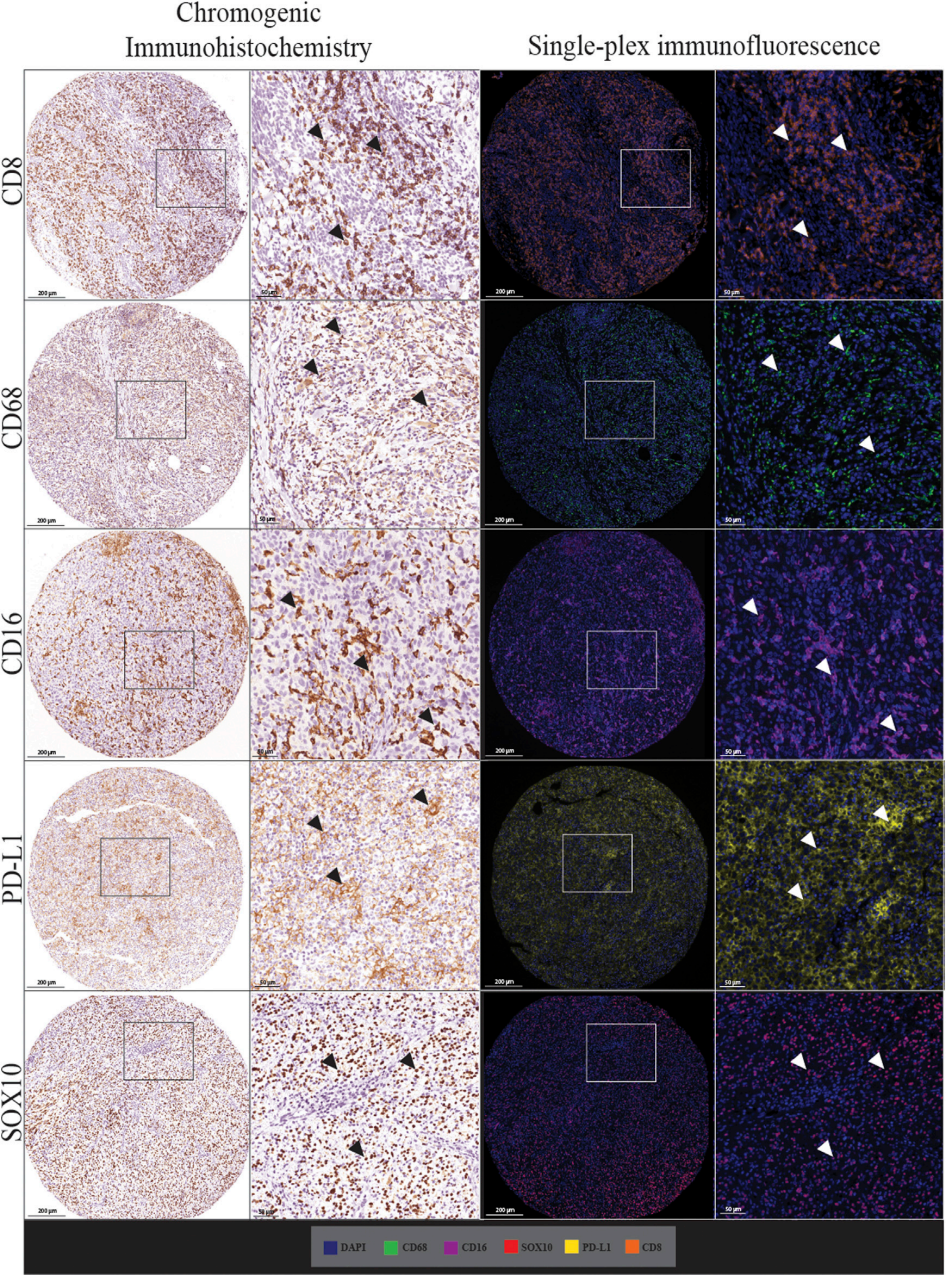
Microphotographs of representative examples of staining with traditional chromogenic immunohistochemistry (left panels) and single-plex immunofluorescence (right panels) along with corresponding 20× sample regions from the same patient core for each marker. White arrows indicate positive staining. Antibodies stained using traditional chromogenic IHC and single-plex immunofluorescence show similar patterns of expression. Scale bars are 200 and 50 µm for the low-magnification (×7.5) and high magnification (×20) microphotographs respectively.

We first assessed whether traditional chromogenic IHC staining corresponded to the single-plex immunofluorescence staining on the TMA and control tissue samples. By comparing the staining pattern in adjacent sections, we found that the two types of staining were comparable as shown in Figure 2. In both IHC and single-plex immunofluorescence, CD8 displayed membranous expression with minimal branches extending from the cellular space into the extracellular region. CD68 was observed in the cytoplasm of macrophages with membranous accentuation, along with some background staining in the extracellular space of the tissue. CD16 displayed cytoplasmic and membranous expression. PD-L1 was identified in the cytoplasm of melanoma cells and macrophages with branches in the cellular membrane. SOX10 expression was localised to the nucleus of melanoma cells with minimal branches into the cytoplasm of cells. SOX10 staining displayed some background across the tissue which was removed upon visual thresholding. The pattern of the stain distribution was either concentrated around the nucleus or diffused in the cytoplasmic space and cellular membrane.

**FIGURE 3:**
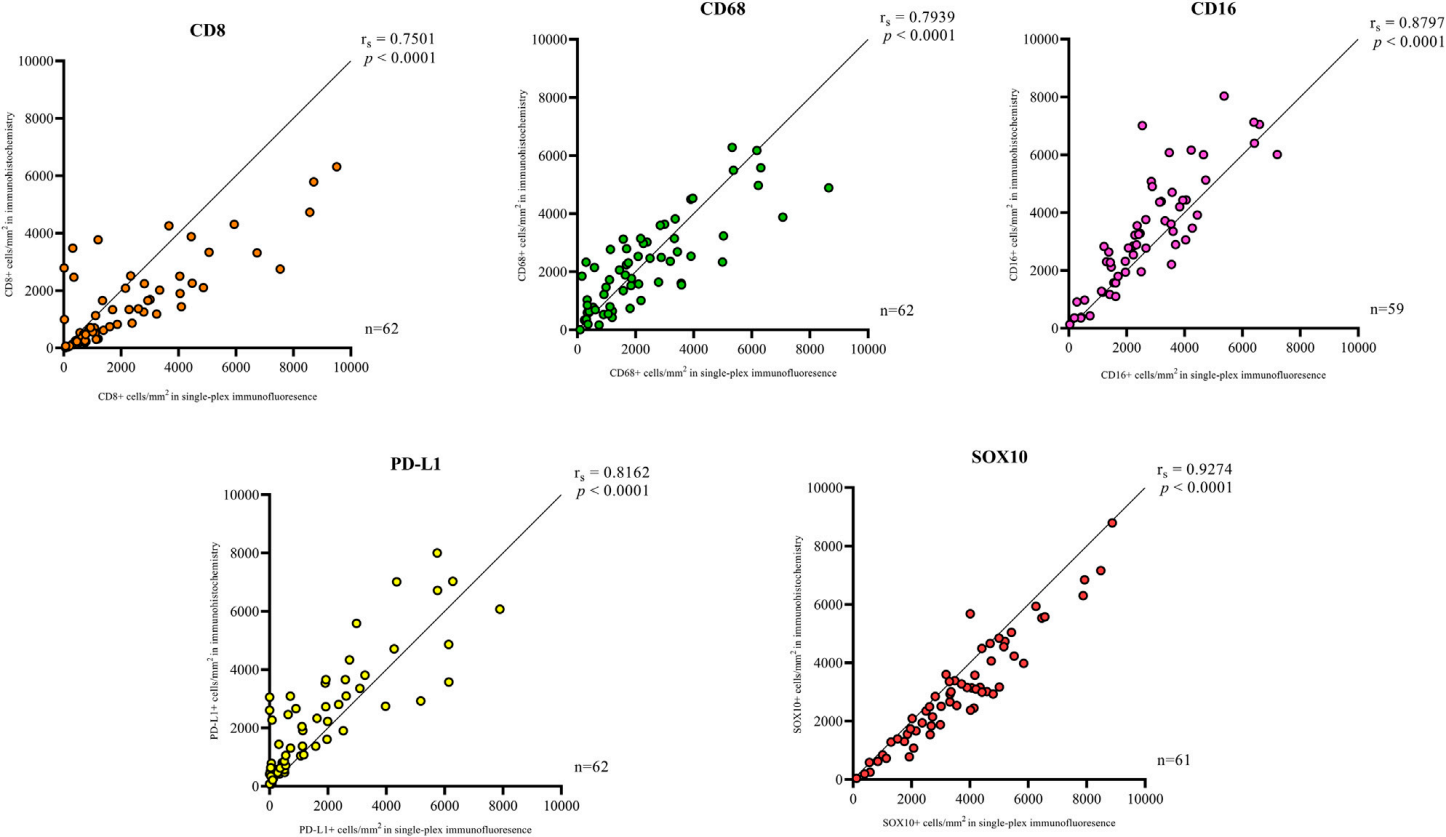
Correlation plots represent the correlation between the density of positive cells in traditional chromogenic immunohistochemistry and single-plex immunofluorescence for each marker in represented. Correlation analysis between the cell densities for each marker showed significant positive correlations. The strongest correlations were observed in CD16 (*n* = 59) and SOX10 (*n* = 61). The lowest correlations were observed in CD8 (*n* = 62). Data are Spearman’s correlation values with *p* < 0.05.

In addition to the staining pattern, we conducted quantification analysis on the entire tissue sections using the Halo image analysis platform and compared the cell densities (positive cell counts relative to the tissue area (mm^2^) for both types of staining across the five markers. The correlation analysis between the cell densities for each marker in chromogenic immunohistochemistry and single-plex immunofluorescence staining showed significant positive correlations, as shown in Figure 3. The strongest correlations were observed in CD16 (*r* = 0.8797, *p* < 0.0001) and SOX10 (*r* = 0.9274, *p* < 0.0001). Correlations between chromogenic immunohistochemistry and single-plex immunofluorescence for CD8 (*r* = 0.7501, *p* < 0.0001), CD68 (*r* = 0.7939, *p* < 0.0001) and PD-L1 (*r* = 0.8162, *p* < 0.0001) were similar.

### Validation of Staining Using Multiplex Immunofluorescence

**FIGURE 4:**
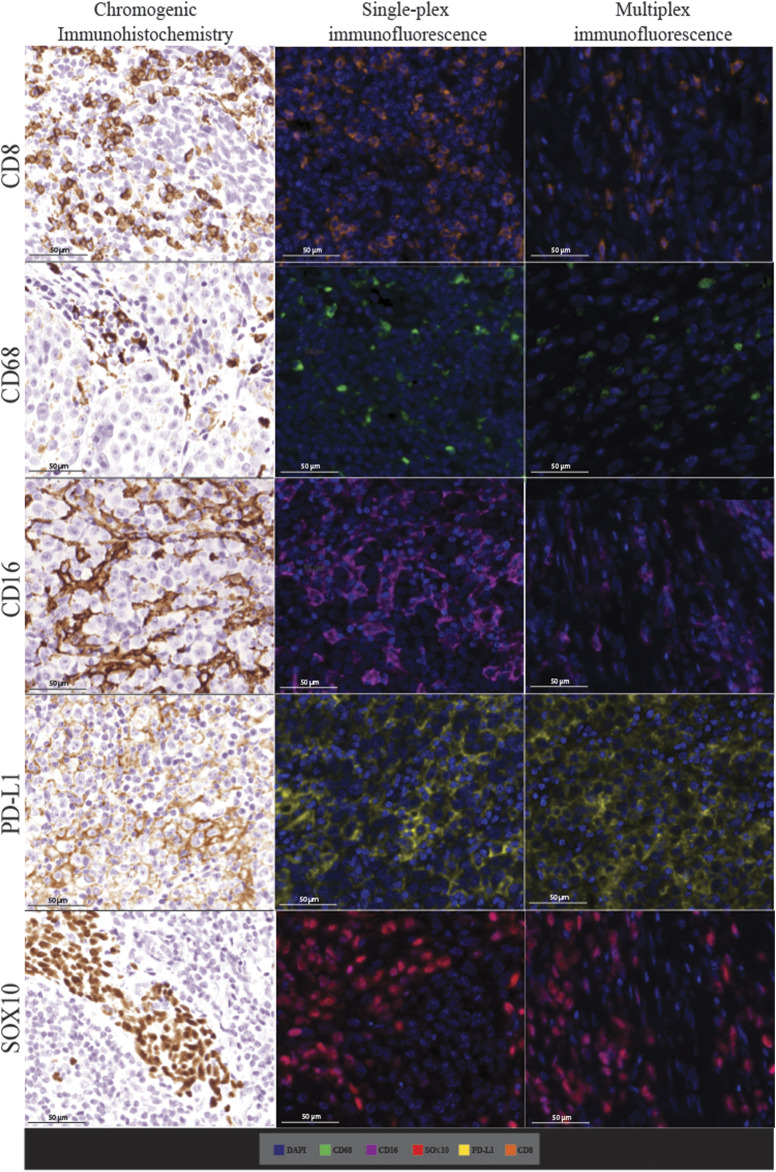
Microphotographs of representative examples of staining in the respective control tissue (a lymph node containing metastatic melanoma) for chromogenic immunohistochemistry (left panels), single-plex immunofluorescence (middle panels), and multiplex immunofluorescence (right panels) display comparable expression patterns for all markers. Scale bars are 50 μm at ×20 magnification.

**FIGURE 5:**
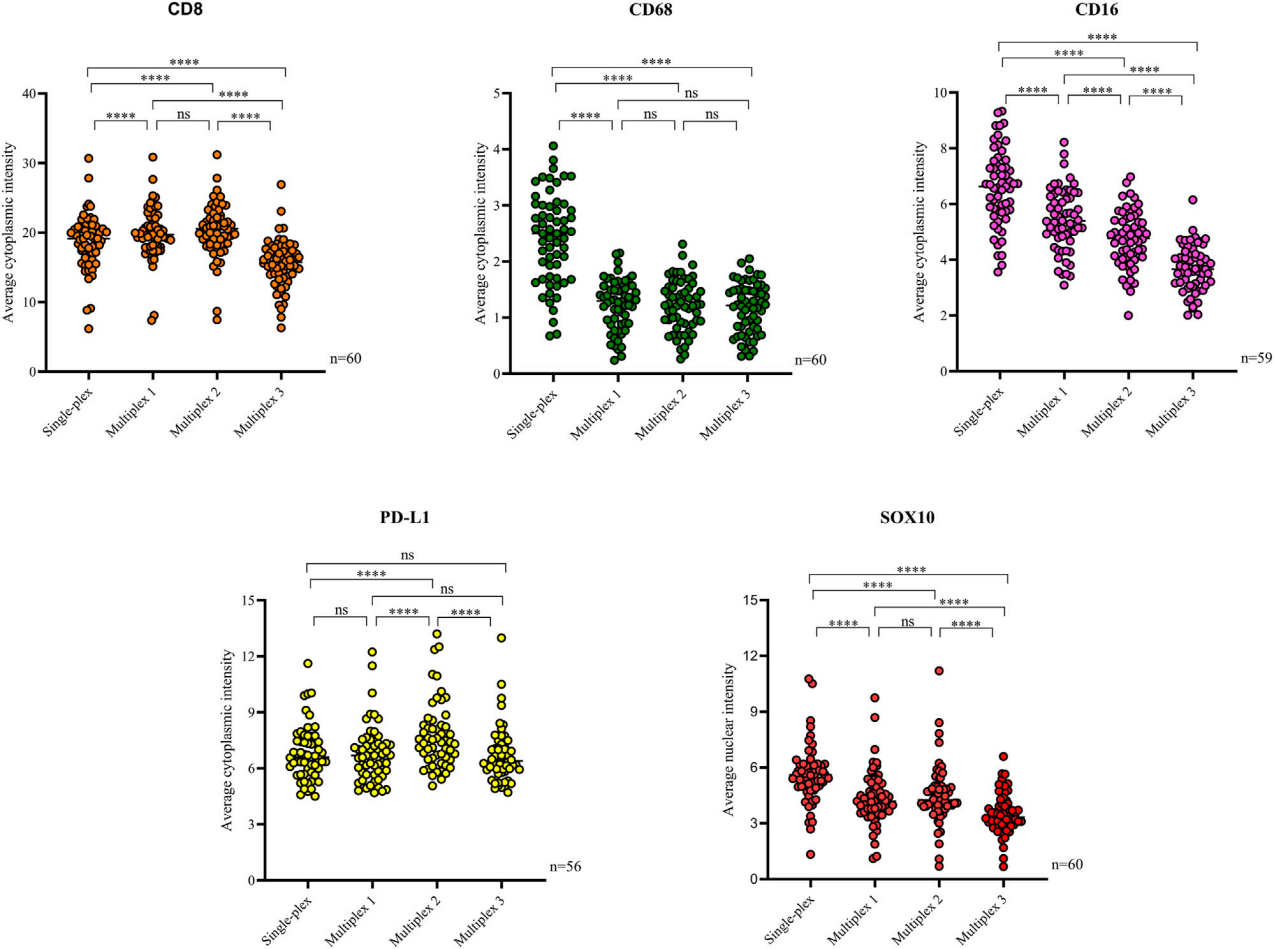
Scatter plots comparing the average intensities of each marker in the single-plex immunofluorescence staining to the intensities in the three multiplex replicates. Single-plex immunofluorescence displayed higher marker intensities for CD68 (*n* = 60), CD16 (*n* = 59) and SOX10 (*n* = 60) compared to the corresponding multiplex immunofluorescence staining. PD-L1 (*n* = 56) single-plex and multiplex immunofluorescence staining demonstrated similar marker intensities. Single-plex immunofluorescence staining with CD68 demonstrated a larger variation in marker intensities compared to the multiplex immunofluorescence intensities. Multiplex 3 displayed lower cell intensities for markers CD8 (*n* = 60), CD16, and SOX10. One-way ANOVA with Tukey’s HSD (honestly significant differences) test, **** = *p* < 0.0001, ns, non-significant.

Following optimisation of the markers using traditional IHC and single-plex staining, we next validated the markers *via* multiplex immunofluorescence. The staining pattern for each antibody was evaluated and compared to the staining on the respective control tissue (Figure 4). Multiplex immunofluorescence staining showed minimal cross-bleed across channels. Marker intensity variation for single-plex and multiplex immunofluorescence staining was observed. Single-plex immunofluorescence displayed higher marker intensities for CD68, CD16 and SOX10 compared to the corresponding multiplex immunofluorescence staining (*p* < 0.0001) (Figure 5). Furthermore, a larger variation in cell intensities was observed in single-plex immunofluorescence staining compared to the multiplex immunofluorescence intensities. Multiplex 3 displayed lower cell intensities for markers CD8, CD16, and SOX10 compared to Multiplex 1 and 2 (*p* < 0.0001). The occurrence of false negative and false positive cells were manually counted for all the markers stained with traditional immunohistochemistry and multiplex immunofluorescence and no significant differences were observed between the two methods (Supplementary Figure S2).

**FIGURE 6:**
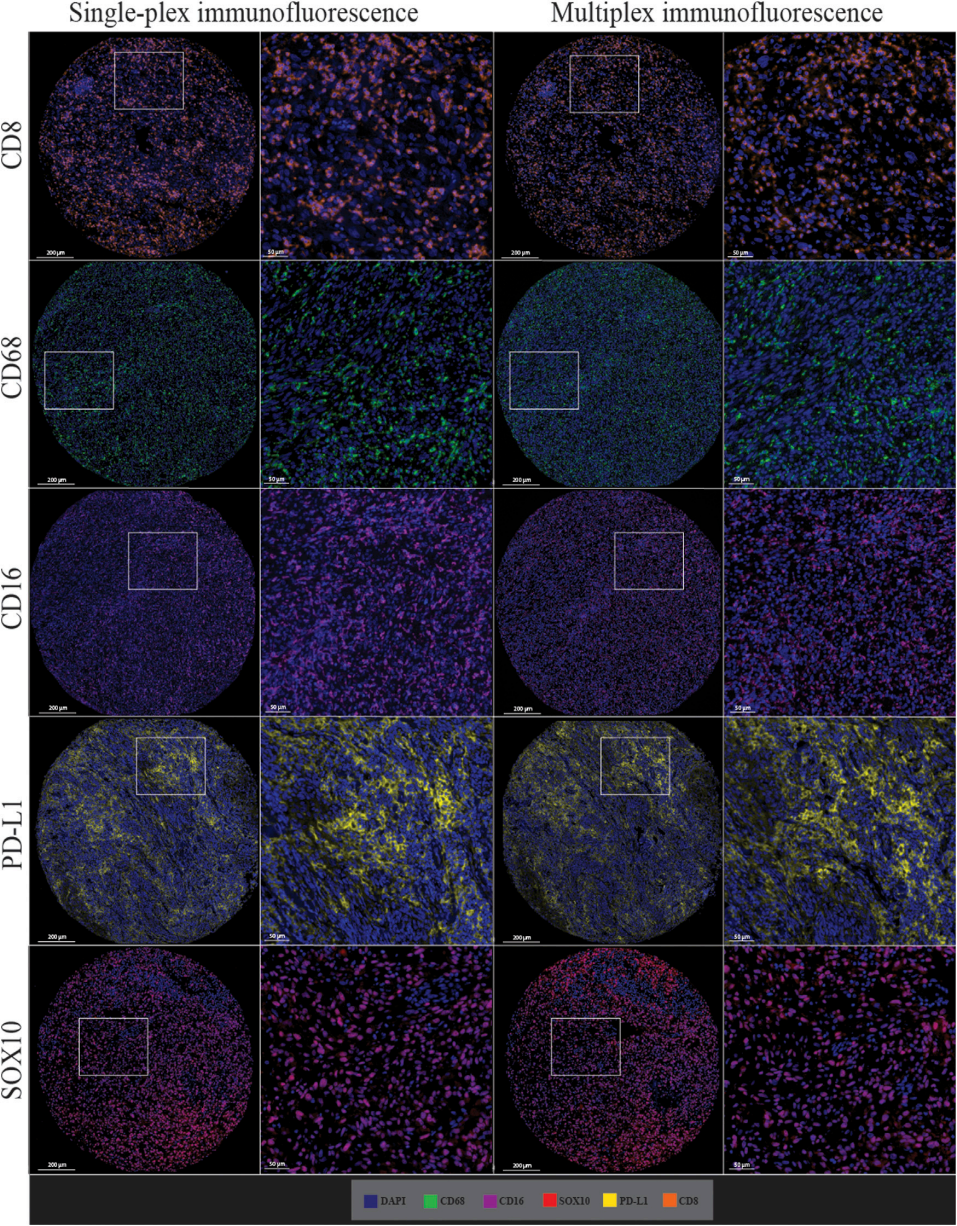
Microphotographs of representative examples of staining in the single-plex immunofluorescence (left panels) and the multiplex immunofluorescence (right panels) along with corresponding ×20 sample regions from the same patient core for each marker. Antibodies stained with single-plex immunofluorescence and multiplex immunofluorescence show similar patterns of expression. Scale bars are 200 and 50 µm for the low-magnification (×7.5) and high magnification (×20) microphotographs respectively.

**FIGURE 7:**
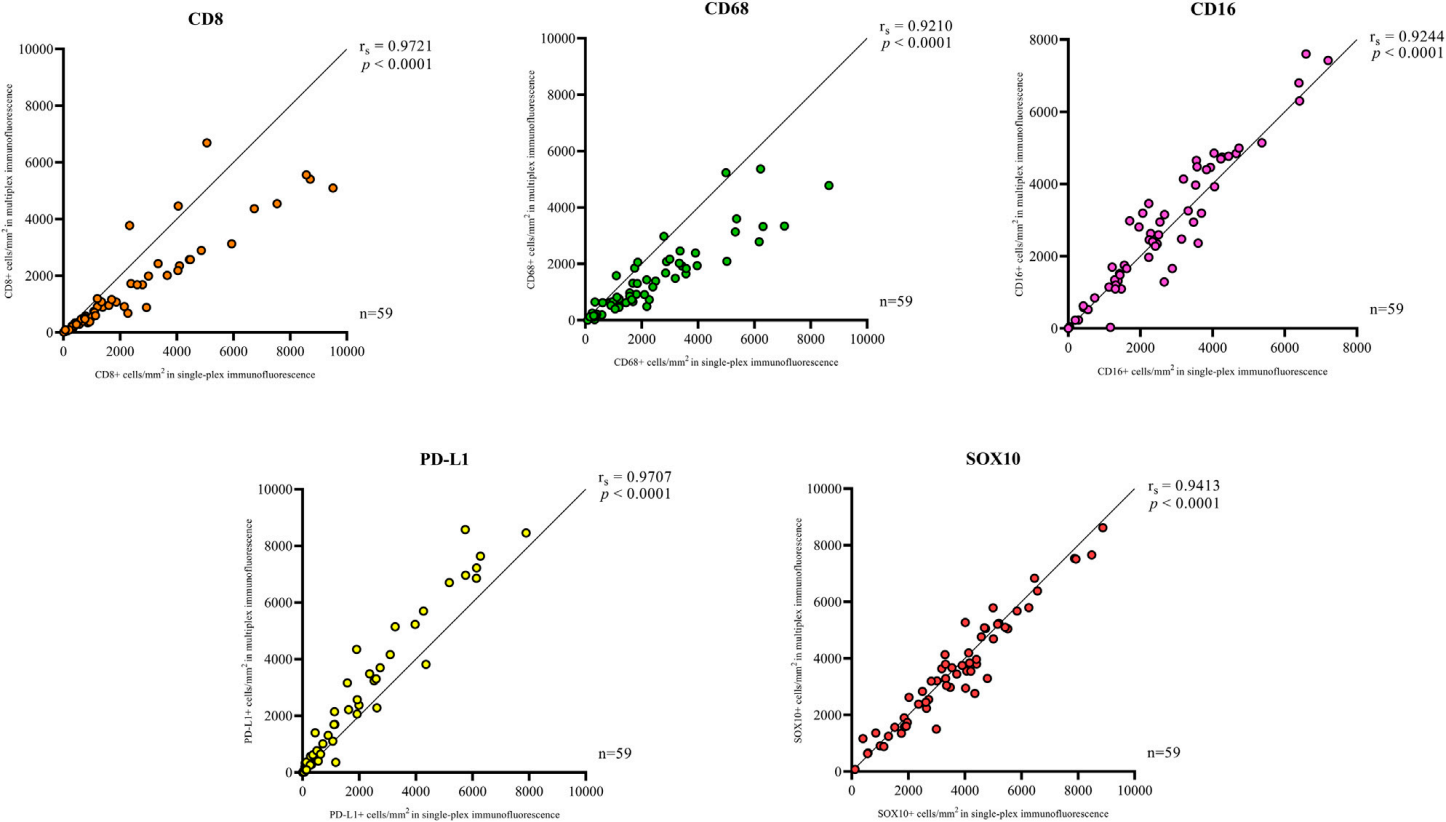
Correlation plots compare the correlation between the cell densities for each marker in single-plex immunofluorescence and multiplex immunohistochemistry. An average of the cell densities of each marker across the three multiplex immunofluorescence sections was used to compare with the single-plex immunofluorescence marker cell density. Significant positive correlations between the single-plex immunofluorescence cell densities and the average cell density of the multiplex immunofluorescence were observed for all markers. CD8 displayed the strongest correlation between single-plex immunofluorescence and multiplex immunofluorescence. Data are Spearman’s correlation values with *p* < 0.05.

Single-plex immunofluorescence staining displayed a similar pattern of staining to that observed in the individual markers in the multiplex panel (Figure 6). The correlation analysis between the cell densities for each marker in single-plex immunofluorescence and the averaged cell density for each marker across the three multiplex immunofluorescence staining showed significant positive correlations for CD68 (*r* = 0.9210, *p* < 0.0001), CD16 (*r* = 0.9244, *p* < 0.0001), PD-L1 (*r* = 0.9707, *p* < 0.0001) and SOX10 (*r* = 0.9413, *p* < 0.0001), as shown in Figure 7. CD8 displayed the strongest correlation between the single-plex immunofluorescence and the average cell density of the multiplex immunofluorescence (*r* = 0.9721, *p* < 0.0001).

### Multiplex Immunofluorescent Staining Displays Reproducibility

**FIGURE 8:**
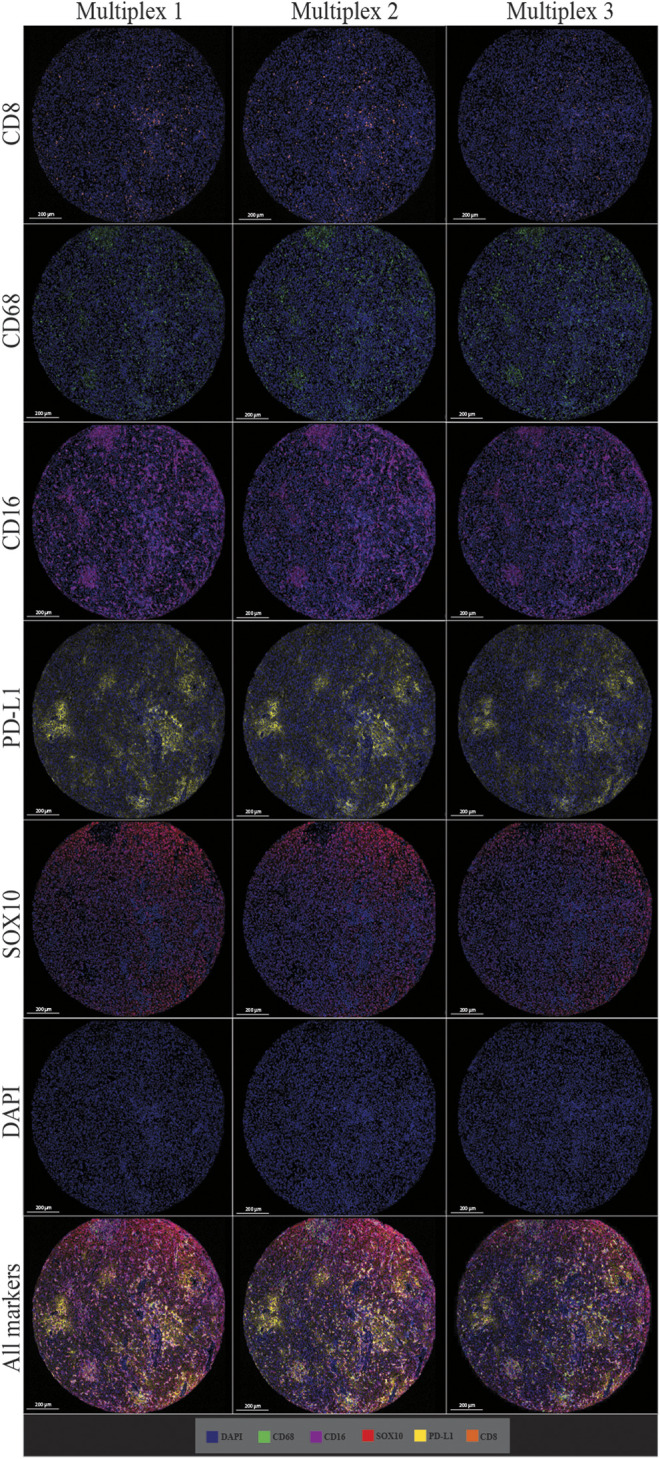
Microphotographs of representative examples of staining in multiplex immunofluorescence cores from the same patient core for each marker show similar patterns of expression in each multiplex. Composite images show the integration of markers on a single slide. Scale bars are 200 μm at ×7.5 magnification.

**FIGURE 9:**
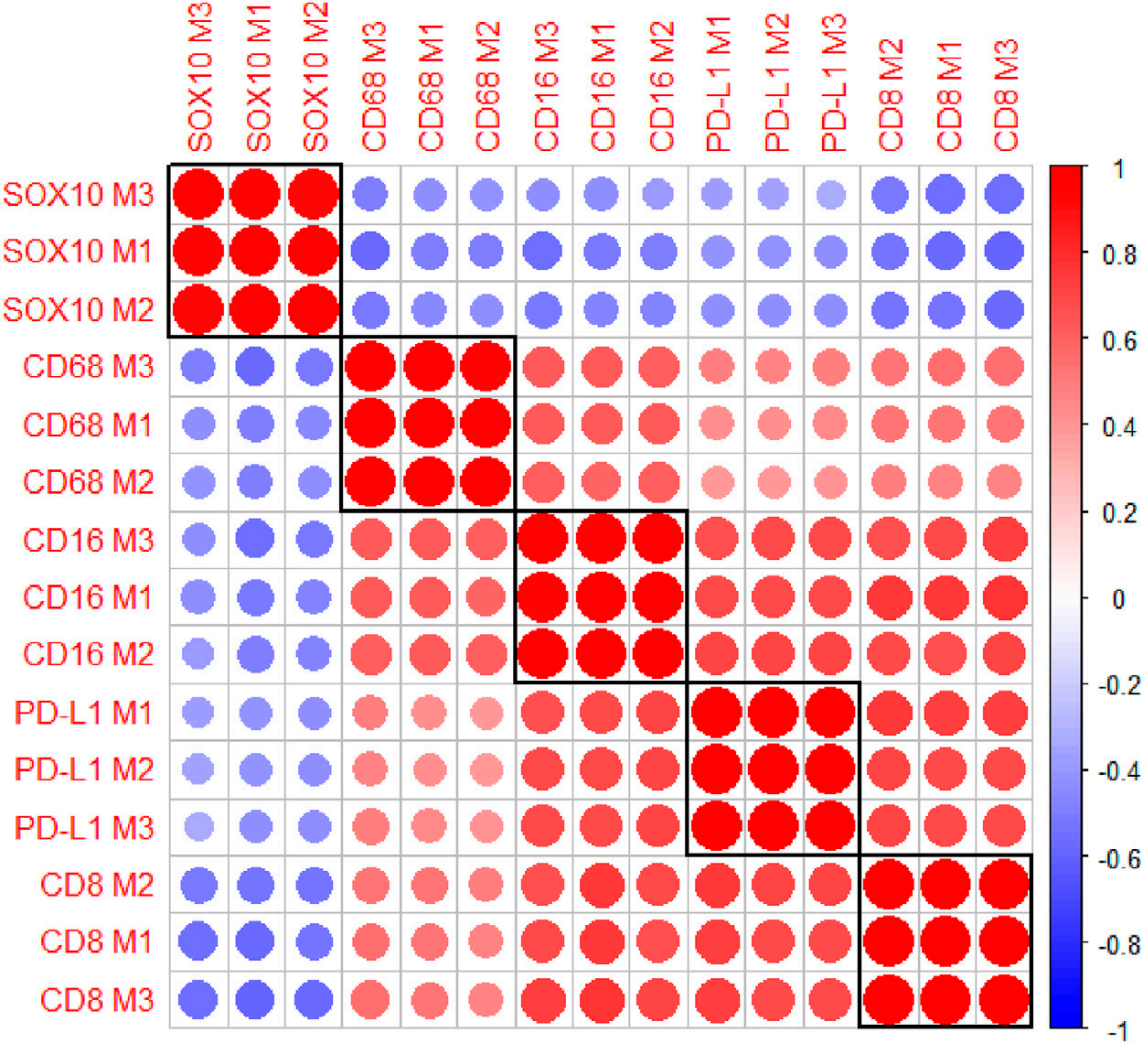
A correlation matrix heatmap comparing the relationship between the cell densities of each marker across the three consecutive multiplex immunofluorescence sections. Highly significant positive correlations were observed between the three Multiplexes for all markers. The strongest correlations were observed for PD-L1 between Multiplex 2 and Multiplex 3. The size of the circles represent the absolute value of the correlation coefficient. The colours of the circles represent the sign and magnitude of the correlation with shades of red representing positive and higher correlation coefficients and shades of blue representing negative and lower correlation coefficients. Data are Spearman’s correlation values with *p* < 0.05.

The multiplex immunofluorescence panel was stained on three consecutive TMA sections to assess the reproducibility of the staining (Figure 8). Highly significant positive correlations were observed between Multiplex 1 and 2, Multiplex 2 and 3, and Multiplex 1 and 3 for all 5 markers (*r* > 0.940, *p* < 0.0001). The strongest correlations between the multiplex batches were observed for PD-L1, particularly between Multiplex 2 and Multiplex 3 (*r* = 0.988, *p* < 0.0001). The lowest correlation was observed for SOX10 between Multiplex 2 and Multiplex 3 (*r* = 0.940, *p* < 0.0001) (Figure 9).

### Co-Localisation Patterns in Multiplex Immunofluorescence

**FIGURE 10:**
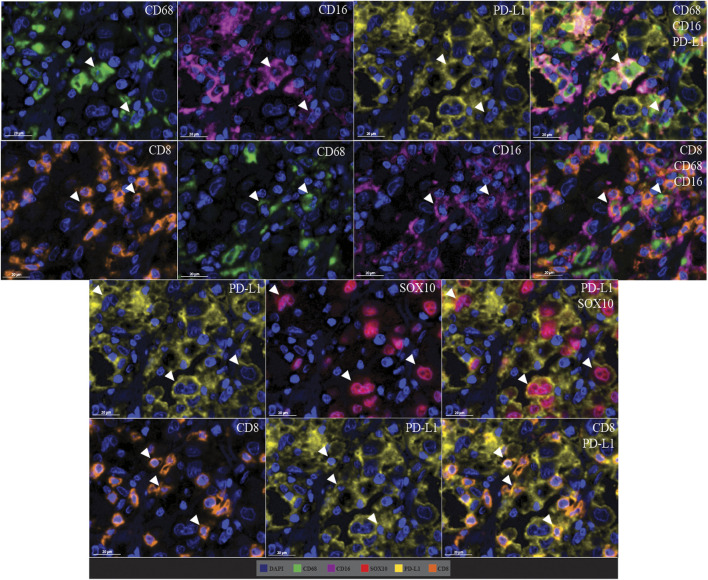
Microphotographs of representative examples of marker co-localisation in multiplex immunofluorescence, showing specific cell phenotypes. Composite images show the integration of markers on a single slide. White arrows indicate cell phenotypes which include PD-L1+ macrophages (CD68 positive, CD16 positive, and PD-L1 positive cells), CD8-expressing macrophages (CD68 positive, CD16 positive, and CD8 positive cells), PD-L1+ tumour cells (PD-L1 positive and SOX10 positive cells), and PD-L1+ cytotoxic T-cells (CD8 positive and PD-L1 positive cells). Scale bars are 20 µm (40×).

Cell co-localisation was also observed in the multiplex immunofluorescence panel, showing specific cell phenotypes. This included PD-L1+ macrophages (CD68 positive, CD16 positive, and PD-L1 positive cells), CD8-expressing macrophages (CD68 positive, CD16 positive, and CD8 positive cells), PD-L1+ tumour cells (PD-L1 positive and SOX10 positive cells), and PD-L1+ cytotoxic T-cells (CD8 positive and PD-L1 positive cells; Figure 10).

## Discussion

Multiplex immunofluorescence and image analysis offers the ability to rapidly and reproducibly quantify cellular populations within tumour biopsies. In this study, we validated a multiplex immunofluorescence panel of immune cell markers CD8, CD68, CD16, the immune checkpoint PD-L1, and melanoma tumour marker SOX10 with single-plex immunofluorescence staining using the Opal workflow along with the golden standard traditional chromogenic immunohistochemistry in a melanoma tissue microarray. To the best of our knowledge, this is the first study to perform such a comparison and is critical for the field, particularly if multiplex immunofluorescence is to be used in clinical practice. Previous studies have qualitatively assessed the comparability of staining for any given antibody in a multiplex protocol relative to a single-plex protocol (Carvajal-Hausdorf et al., 2015). Recent studies have also quantitatively compared the accuracy of multiplex and single-plex staining (Parra et al., 2017). In the present study the expression of the markers was quantified and compared across all three staining categories, showing accurate and reproducible results. The multiplex protocol was fine-tuned at several steps, particularly the concentrations and incubation times of primary antibodies, to ensure sensitivity and optimal detection, as reported previously (Syed et al., 2019). Based on our data, we can conclude that multiplex immunofluorescence is an invaluable tool for accurately profiling the tumour microenvironment by detecting the expression and cellular distribution of several immune targets in a single tissue while preserving tissue architecture. This is particularly useful in identifying predictive biomarkers for patients receiving immunotherapy but before this technique can be routinely used in diagnostic pathology, the automated protocols needed to be optimised and validated, as we have shown here.

The multiplex staining in this study was conducted using the TSA methodology using Opal reagents. TSA is reputable due to its high sensitivity and specificity in identifying markers, particularly low-expressing targets. It is also advantageous in its ability to reduce the potential for antibody cross-reactivity (Lim et al., 2018). However, the advantageous nature of its sensitivity could also contribute to greater levels of unspecific background staining. Apart from CD16a, the antibody incubation time and primary antibody concentrations using the TSA methodology was generally shorter than the chromogenic IHC due to the signal amplification and enhancement. While we observed significant positive correlations between cell densities of markers in chromogenic immunohistochemistry and Opal staining, both with the single-plex and multiplex immunofluorescence staining, the correlation was lower for the chromogenic staining compared to single-plex and multiplex immunofluorescence staining. The difference observed could be attributed to signal amplification in the Opal staining by using the TSA methodology or the digital unmixing of hematoxylin and DAB in brightfield images. Variability in cytoplasmic and nuclear intensities for the markers stained using single-plex immunofluorescence and multiplex immunofluorescence was observed among the four groups, particularly for CD68, CD16 and SOX10 which all had higher single-plex cell intensities compared to multiplex replicates. This variability is possibly attributed to the serial stripping and re-staining along with sample-to-sample and batch-to-batch variation. Since CD68, CD16 and SOX10 were the first three markers in the multiplex panel, they may have been more affected by the serial tripped compared to the PD-L1 and CD8 markers. This was accounted for by tailoring the analysis algorithms to ensure adequate marker detection for all samples. Despite the variation in staining intensity, the cell density of markers in both staining types exhibited significant positive correlations with little disparity. Therefore, translation from chromogenic to immunofluorescence staining is highly correlated, but may require validation for clinical purposes. Translations from single-plex to multiplex immunofluorescence staining is also highly correlative, and adjustment for a reduction in staining intensity in multiplex techniques is useful.

While traditional chromogenic immunohistochemistry and multiplex immunofluorescence have been used for decades in clinical and research settings, their ability to assess only a small selection of markers at a given time limits their scope in profiling the TME. Advances in multiplex fluorescence imaging technologies have drastically and rapidly improved our ability to characterise the TME at the single-cell level by increasing the number of markers detectable in a single tissue. Highly multiplex fluorescence based systems, such as, the MACSima Imaging Cyclic Staining (MICS) can detect hundreds of fluorescently-tagged antibodies at a sub-cellular level in a single tissue section through the use of automated cycles of sequential staining, image acquisition, and signal erasure through photobleaching the fluorescent labels of the antibodies (Kinkhabwala et al., 2021). Similarly, the co-detection by indexing (CODEX) platform also uses iterations of staining and removing. However, it does not add and remove the antibody but uses a complementary fluorescently labelled DNA probe that binds to the antibodies conjugated with DNA oligonucleotides (Black et al., 2021). The additional and removal of fluorescently-tagged components overcomes the limitation of spectral overlap which can be an issue in standard multiplex immunofluorescence staining and other multi-round immunofluorescent staining techniques (Zielinski et al., 2021). While new technologies like MICS and CODEX broaden the scope of analysis and offer promising contributions for use in personalised medicine, these technologies have not been widely used and validated as traditional chromogenic immunohistochemistry and multiplex immunofluorescence.

Clinical utility of immunohistochemistry is highly reliant on the appropriate validation of the primary antibodies. For this study, each marker required extensive wet-lab optimisation for all staining types on control tissue to guarantee reproducible staining and antibody functioning. All antibodies included in our panel were initially confirmed using chromogenic immunohistochemistry on control tissue following manufacturer recommendations and assessed by clinical pathologists to ensure the antibody was staining as expected and to note the expected staining pattern for each marker. Optimisation of the single-plex and multiplex immunofluorescence staining involved titrating the primary antibody on control tissue, testing different pairings of primary antibodies and Opal fluorophores, and testing different sequences of antibodies to gauge the epitope stability with various antigen retrieval cycles and ensure sensitivity and specificity (Syed et al., 2019). Automated staining is advantageous for many reasons, mostly in terms of speed, reproducibility, and reduction in human error. Despite this, there is the possibility of errors like staining gradients across slides due to precise mechanical drop placements and the positioning and size of tissue (Rolls et al., 2008). This error was not observed in the current study as tissue sections were mounted with the boundaries of drop placements in mind. Other staining artifacts like tissue distortion or pigment accumulation inherent to all staining workflows were excluded from analysis.

Image analysis relies heavily on clean staining patterns with high signal to noise ratios. In our high-resolution multispectral images, we did not see significant non-specific background staining except for CD68 and, to a lesser extent, PD-L1. PD-L1 is reported to exhibit staining in the cytoplasm, cell membrane and inflammatory infiltrate with different levels of intensity (Cedrés et al., 2015). Distinguishing between varied PD-L1 expression and potential non-specific background staining proved to be challenging. Along with the other markers, PD-L1 staining was reviewed by a pathologist to ensure the staining pattern observed contained minimal background staining. While CD68 displayed a diffuse staining pattern branching out from the cytoplasmic region along with staining lysosomes, as reported previously (Chistiakov et al., 2017). This similarly posed a challenge in identifying the margins of a given cell expressing CD68 staining, especially when delineating CD68 expression in neighbouring cells. We compared CD68 expression in our current staining with our previous staining and the manufacturer’s guidelines to ensure our identification of CD68 was accurate. SOX10 exhibited a clear nuclear staining pattern with minimal leak into the cytoplasmic region of a cell and served as a useful marker in identifying tumour cells, as reported previously (Mohamed et al., 2013). CD16 and CD8 displayed clear staining in the cytoplasmic and membranous cellular space respectively and relatively compactly (Stasikowska-Kanicka et al., 2018; Steele et al., 2018). All markers were visually thresholded multiple times in a systematic manner to ensure any level of residual background staining was filtered out and expression intensity of cells definitively positive was used to set the threshold for each marker. This was conducted for each marker in each staining category along with separate thresholds established for the three multiplex slides to ensure batch to batch variation was accounted for.

## Conclusion

To conclude, we have validated the accuracy of an automated multiplex immunofluorescence panel designed to analyse and phenotype melanoma and immune cells in the tumour microenvironment at single-cell resolution. We demonstrated reproducible staining with the accurate detection of immune cell markers CD8, CD68, CD16, immune checkpoint PD-L, and melanoma tumour marker SOX10 in a multiplex methodology compared with chromogenic immunohistochemistry and single-plex immunofluorescence staining for all markers. The accurate and reproducible nature of multiplex immunofluorescence provides confidence that this technique can be used to develop panels including other targets and explore opportunities for innovative digital image analysis approaches such as spatial interactions between markers of interest, inflammatory tumour infiltration, phenotyping, and predictive biomarker assessment. This validated workflow allows us to obtain high-quality staining data and is particularly useful in immunology studies and biomarker development.

## Data Availability

The original contributions presented in the study are included in the article/Supplementary Material, further inquiries can be directed to the corresponding author.
