# Supplementary material for: Validation of an Accurate Automated Multiplex Immunofluorescence Method for Immuno-Profiling Melanoma
Source: Front Mol Biosci. 2022 May 19;9:810858. doi: 10.3389/fmolb.2022.810858 (PMC9160303; doi:10.3389/fmolb.2022.810858)
Supplement: Supplementary file 1 [file DataSheet1.PDF]

SUPPLEMENTARY TABLES AND FIGURES

Supplementary Table 1: The signal-to-noise ratio (SNR) from 3 cores from each staining category was calculated based on visual confirmation of signal and noise localisation. The mean grey value was determined by generating intensity profiles for both signal and noise in FIJI (ImageJ), and the SNR was calculated as: average of local maximum values from the signal intensity profile divided by average of all intensity values in the noise intensity profile.

	**CD68**	**CD16**	**SOX10**	**PD-L1**	**CD8**
**IHC1**	14.6697	30.26481	29.55033	11.77701	51.63297
**IHC2**	12.14103	34.04018	10.45018	10.6863	22.74116
**IHC3**	35.65987	30.77555	18.67017	13.84266	31.06458
**SP1**	132.4178	52.00513	25.69085	31.1863	157.6198
**SP2**	44.10719	123.5216	11.71931	27.31187	121.2176
**SP3**	111.0561	205.1733	12.02886	13.0331	42.29162
**M1_1**	14.80093	37.88032	26.74101	13.15969	20.31067
**M1_2**	16.35975	16.45087	92.6757	10.32674	21.44686
**M1_3**	23.54609	13.92454	51.6041	16.21186	18.59181
**M2_1**	24.95164	18.24809	91.31181	16.10499	23.90259
**M2_2**	11.16472	30.45261	33.69124	16.82591	31.8693
**M2_3**	28.72388	16.06082	49.59614	53.217	24.90751
**M3_1**	25.18109	13.54822	23.62147	14.65791	25.60351
**M3_2**	12.04482	20.02584	16.46304	10.09323	34.09671
**M3_3**	12.85234	16.54018	37.02821	14.28172	13.37006

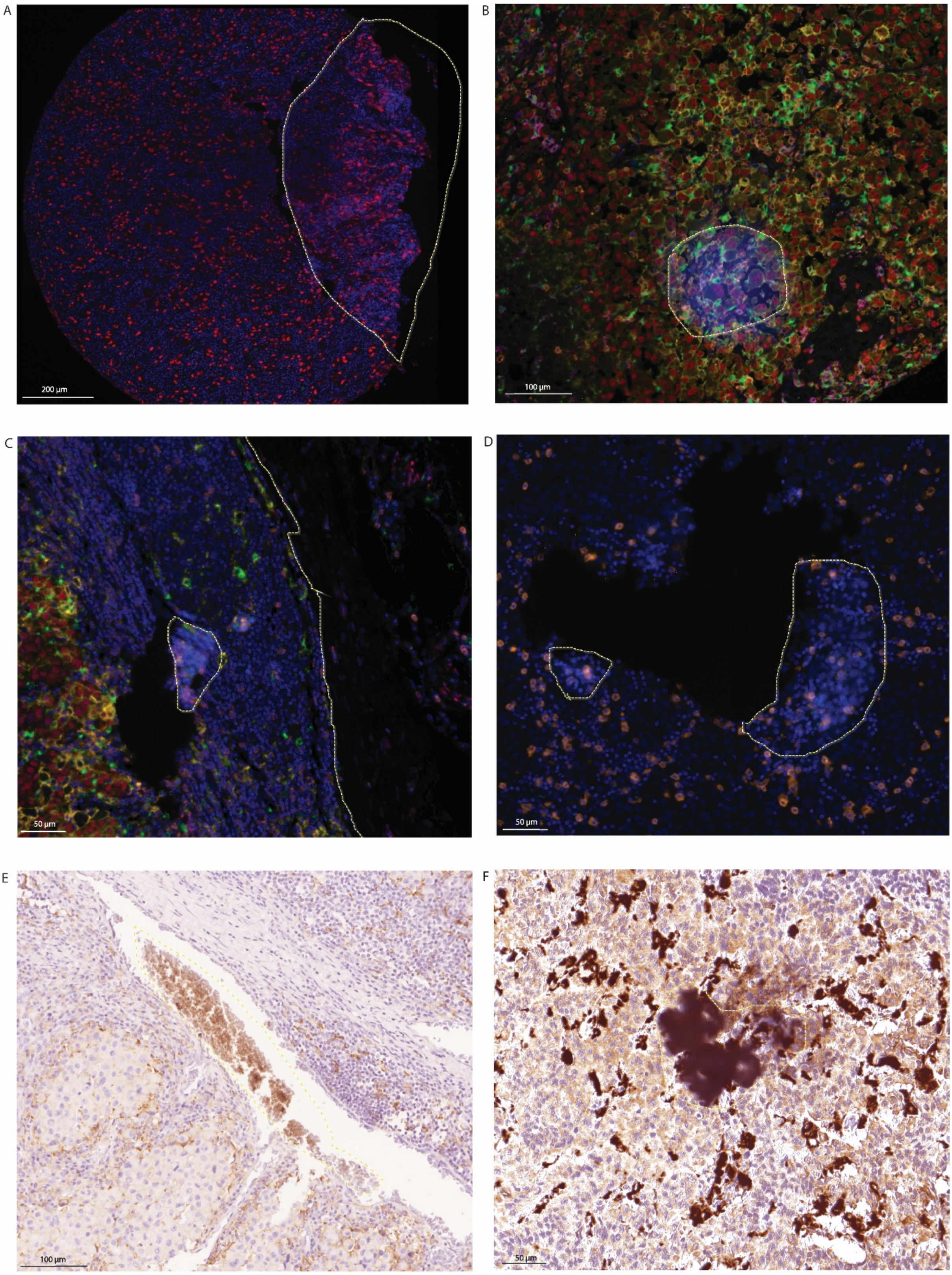

Supplementary Figure 1: Microphotographs showing the following staining artifacts: (**A**) Tissue fold, (**B**) Lens glare, (**C-D**) compressed or crushed tissue, (**E-F**) pigment accumulation and non-specific staining. The dotted yellow lines encompass regions excluded from analysis.

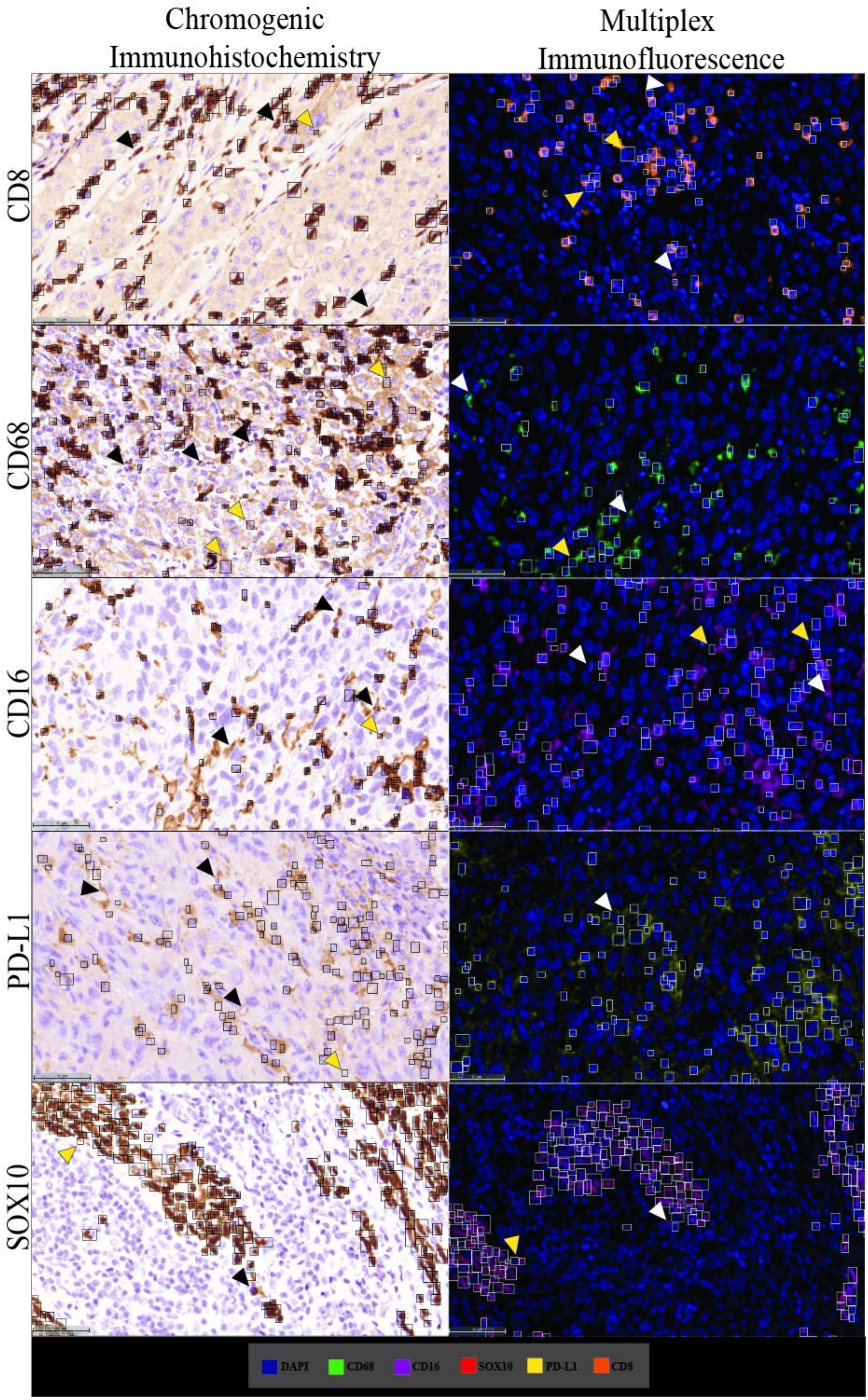

Chromogenic Immunohistochemistry
Multiplex Immunofluorescence
CD8
CD68
CD16
PD-L1
SOX10
DAPI
CD68
CD16
SOX10
PD-L1
CD8

Supplementary Figure 2A: All markers stained with traditional chromogenic immunohistochemistry and multiplex immunofluorescence at 40x magnification show similar counts of false negatives. Boxes indicate positive cells detected by the analysis settings. Arrows indicate false negatives. Scale bars = 50 µm.

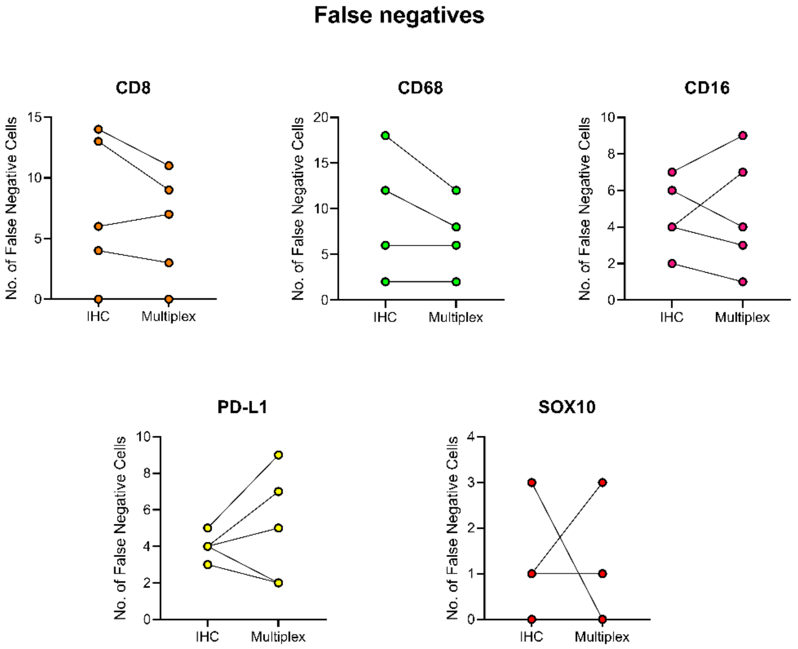

Supplementary Figure 1B: False negatives in 5 regions (40x magnification) were counted for all the markers stained with traditional IHC and multiplex immunofluorescence. No significant differences were observed between the staining categories. Wilcoxon test, $p<0.05$.

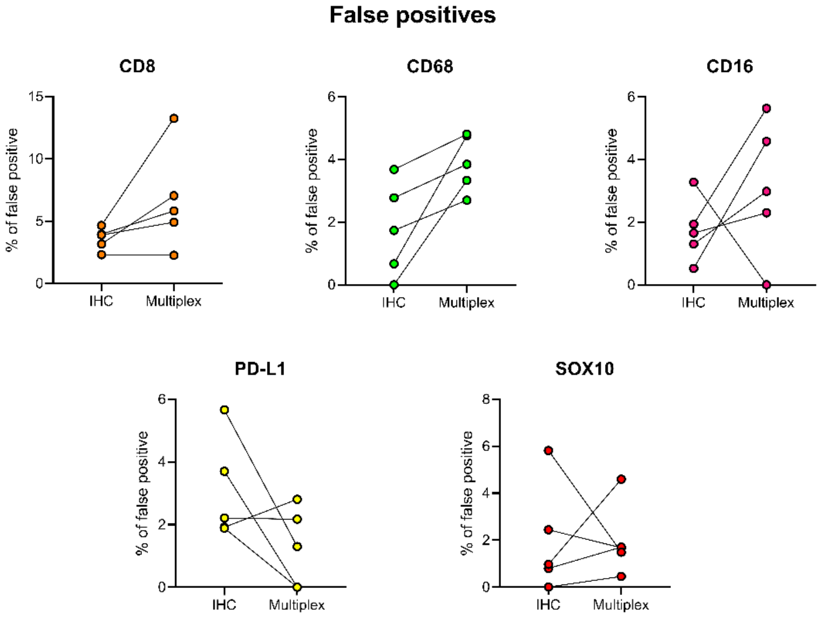

Supplementary Figure 2C: The percentage of false positives in the total number of positive cells in 5 regions (40x magnification) were counted for all the markers stained with traditional IHC and multiplex immunofluorescence. No significant differences were observed between the staining categories. Wilcoxon test, $p<0.05$.
